# CD11b/CD86 involved in the microenvironment of colorectal cancer by promoting Wnt signaling activation

**DOI:** 10.1002/cam4.70245

**Published:** 2024-09-20

**Authors:** Junyu Ke, Guirong Chen, Yihui You, Qinghua Xie, Zheng‐lin Liu, Chunhui Song, Yanqiu Zheng, Zejun Shan, Jinbin Song, Zhangyu Jiang, Haiyan Wang, Qun Du, Yongqiang Wu, Xin‐lin Chen, Yanwu Li

**Affiliations:** ^1^ School of Basic Medical Sciences Guangzhou University of Chinese Medicine Guangzhou China; ^2^ Gaozhou Hospital of Traditional Chinese Medicine Affiliated to Guangzhou University of Chinese Medicine Gaozhou China; ^3^ Science and Technology Innovation Center Guangzhou University of Chinese Medicine Guangzhou China; ^4^ Maoming Hospital of Traditional Chinese Medicine Affiliated to Guangzhou University of Chinese Medicine Maoming China; ^5^ Animal Experiment Center Guangzhou University of Chinese Medicine Guangzhou China; ^6^ The First Clinical Medical College of Guangzhou University of Traditional Chinese Medicine Guangzhou China; ^7^ Guangzhou International Bio Island Guangzhou China; ^8^ International Institute for Translational Chinese Medicine Guangzhou University of Chinese Medicine Guangzhou China

**Keywords:** colitis associated colon cancer, colorectal cancer, tumor microenvironment, tumor stem cell, tumor‐associated macrophage, Wnt signaling pathway

## Abstract

**Background:**

Colorectal cancer (CRC) is a malignancy that arises within the gastrointestinal tract. Despite ongoing research, the etiology and pathogenesis of CRC remain elusive; particularly, the distribution and characteristics of tumor‐associated macrophages is currently an active area of investigation in understanding the pathological progression and prevention of CRC.

**Methods:**

This study utilized CRC patient surgical samples, mouse models of colitis‐associated cancer, colonic organoid, and co‐culture cell line to examine the changes in CD11b/CD86 at different pathological region and detect the Wnt signaling pathway activity.

**Results:**

Our findings revealed a sensitive and increased expression of CD11b from the early to the advanced CRC tissues and correlated with poor prognosis, while CD86 expression was reduced in advanced CRC tissues. CD133 expression was also elevated in advanced CRC tissues and mainly co‐localized with CD11b, suggesting a positive regulatory effect of CD11b and CD133 expression that may contribute to CRC progression. In AOM/DSS mouse models, activation of the Wnt signaling pathway was associated with increased CD133 and CD11b expression. In vitro, THP‐1 cell was induced to high expression of CD11b, and the above conditional cultural medium enhanced HCT116 cell colony number and CD133 protein expression. Furthermore, colonic crypts from AOM/DSS mouse models were isolated to culture, and the colonic organoids exhibited dilation and significant increases expression of CD133 and β‐Catenin/N‐P‐B‐Catenin.

**Conclusions:**

CD11b might be an important factor to participate the progress of CRC. And the high CD11b of CRC microenviroment might potentially promote CD133 expression and associate with Wnt signal activation.

## 1 INTRODUCTION

Colorectal cancer (CRC), a malignancy affecting the digestive system, is characterized by a high incidence and fatality rate.^1^ During tumor progression, the tumor microenvironment (TME) plays a crucial role in facilitating the initiation and spread of tumors, promoting angiogenesis, enhancing tumor cell proliferation, suppressing tumor cell apoptosis, interfering with growth inhibitory signals, and inducing various dysfunctions.^2^, ^3^ Tumor‐associated macrophages (TAMs) are key constituent of the TME, typically categorized as either classically activated M1 macrophages or alternatively activated M2 macrophages.^4^ M1 macrophages are known to secrete pro‐inflammatory cytokines including tumor necrosis factor (TNF)‐α, interferon (IFN)‐γ, and CXCL‐10, while also producing elevated levels of iNOS, CD86, and CD80. Conversely, M2 macrophages are characterized by the secretion of anti‐inflammatory cytokines such as IL‐4, IL‐10, and TGF‐β, along with high expression of arginase‐1, CD11b, and CD206.^5^, ^6^ Both M1 and M2 macrophages exhibit a high plasticity in response to alterations in the tumor microenvironment or therapeutic interventions. Moreover, TAMs have been shown to influence the survival, self‐renewal, and resistance to chemotherapy of cancer stem cells (CSCs) through the secretion of diverse cytokines. Conversely, CSCs can also attract macrophages by releasing various cytokines and chemokines, prompting their differentiation into TAMs. This dynamic interplay between TAMs and CSCs contributes significantly to tumor progression.

The Wnt signaling pathway is integral in various biological processes, including body growth, development, metabolism, and stem cell maintenance, with its dysregulation being closely associated with cancer and other malignant diseases.^7^, ^8^ Additionally, the Wnt signaling pathway is implicated in both the modulation of macrophage differentiation and function. Several studies have demonstrated that macrophages play a crucial role in promoting the differentiation of macrophages to the M2a phenotype through the activation of the Wnt/β‐catenin signaling pathway, thereby facilitating the repair of mouse gastric mucosa.^9^, ^10^ Conversely, the upregulation of Wnt5a in gastric mucosa has been shown to attract macrophages and stimulate their activation, leading to the release of pro‐inflammatory factors such as TNF‐a and cyclooxygenase‐2 (COX‐2), exacerbating stomach inflammation. Additionally, Zhang et al. found that dextran sulfate sodium (DSS) can induce colitis by triggering macrophage activation and activating the Wnt/β‐catenin signaling pathway.^11^ Activated macrophages facilitate β‐catenin nuclear accumulation and Wnt/β‐catenin signal transduction by inhibiting GSK3β phosphorylation, leading to the induction of gastric mucosal tumors. Additionally, Wnt5a contributes to macrophage aggregation and gastric cancer development through the up‐regulation of MCP‐1.^12^

This study examined colorectal cancer tissue from patients, animal models, cellular levels, and in vitro organoid culture in order to offer novel insights and scientific evidence for the screening and treatment of early‐stage colorectal cancer patients in a clinical context.

## 2 MATERIALS AND METHODS

### 2.1 Patients

A total of 65 CRC patients were admitted to the Second Department of External Medicine at Gaozhou Hospital of Traditional Chinese Medicine between January 2018 and December 2021. Additionally, 27 CRC surgical specimens were utilized for real‐time fluorescence quantification PCR (RT‐qPCR) analysis. All patients were subjected to the TNM staging criteria outlined in the 8th edition published by the American Joint Committee on Cancer (AJCC) and the Union for International Cancer Control (UICC).^13^ The final follow‐up was carried out either in an outpatient setting or via telephone, with the data being cutoff as of December 31, 2022. All colorectal cancer patients signed an informed consent statement.

### 2.2 Experimental animals and the establishment of colitis‐associated cancer (CAC) animal model

Half male and half female SPF C57BL/6 mice (20–22 g, 6–8 weeks old) were purchased from Guangdong Experimental Animal Center. The mice model were induced by AOM/DSS as follow steps: (1) AOM (12 mg/kg) was injected intraperitoneal once. (2) After 6 days of normal feeding, 3% DSS water were given to drink for 5 days and the normal drinking water replaced for 14 days. (3) The step (2) was repeated for three times, with weight monitored every 2 days during DSS treatment. If mice lost more than 10–20% of their body weight, they were given 1 mL sterile saline.

### 2.3 Cell stimulation polarization and conditioned co‐culture

Human monocyte THP‐1 cells were cultured and treated with PMA (100 nmol/L) for 24 h. Subsequently, cells were stimulated with PMA (100 nmol/L) + LPS (100 ng/mL) + IFN‐γ (20 ng/mL) or PMA (100 nmol/L) + IL‐4 (20 ng/mL) + IL‐13 (20 ng/mL) for 36 h to induce polarization and differentiation into a cell model with high CD11b expression. The cells were then divided into THP‐1, PMA + LPS + IFN‐γ, and PMA + IL‐4 + IL‐13 groups. RT‐qPCR was utilized to assess the relative mRNA levels of CD11b in each group. HCT116 was co‐cultured with above induced THP‐1 medium. Cell proliferation was measured at 6, 12, 24, and 72 h using MTT assay. Colony formed were counted and the protein expressions of β‐Catenin, N‐P‐β‐Catenin, Wnt5a, Wnt3a, and CD133 were analyzed by western blot at 72 h.

### 2.4 Organoid culture

At the 10th week of CAC mouse modeling, colon tissues were extracted from each experimental group and longitudinally dissected along the mesentery in a sterile environment. Following cleaning, the colon tissues were sectioned and thoroughly washed with pre‐cooled PBS. The supernatant was then discarded, and 15 mL of gentle cell dissociation reagent was added to the tissues, which were subsequently incubated on a shaker (20 rpm) at room temperature for 15 min. The supernatant was removed, and 10 mL of 0.1% BSA solution (99 mL cold PBS + 1 mL 10% BSA) was added to resuspend the tissue. After standing for 30 s, the supernatant was removed for filtration, with this process repeated 3–4 times. Following centrifugation at 1000 rpm for 5 min, the intestinal stem cells obtained from the precipitation were collected. The precipitates were subsequently re‐suspended in 10 mL of 0.1% BSA solution (99 mL cold PBS + 1 mL 10% BSA), and the quantities and functionalities of the organoids were examined and assessed using a microscope. The suspension was then centrifuged at 1000 rpm for 5 min, the supernatant was decanted, and the remaining solution was combined with 500 μL of Intestinal Mouse Basal Medium and 500 μL of pre‐cooled matrix gel to create a colonic crypt stem cell suspension. This suspension was then dispensed into a 24‐well plate. Cells were incubated at 37°C in a 5% CO_2_ incubator for 15 min. After the matrix glue solidified, 750 μL of Intestinal Mouse Basal Medium was added, and the medium was changed three times a week.

### 2.5 Immunohistochemistry

Following dewaxing, antigen retrieval, and blocking, sections were subjected to overnight incubation with a single‐drop mono‐antibody at 4°C. The following day, a second antibody was added and incubated at room temperature for 1 h, followed by a 20‐min incubation with horseradish‐labeled streptomycin working solution. Subsequently, the sections were stained with DAB chromogenic solution for 3–5 min, followed by a 30‐s hematoxylin stain. Finally, the samples were rendered transparent, air‐dried, sealed with neutral resin, and examined under an optical microscope. Following dewaxing, antigen retrieval, and blocking, sections were subjected to overnight incubation with a single‐drop mono‐antibody at 4°C. The following day, a second antibody was added and incubated at room temperature for 1 h, followed by a 20‐min incubation with horseradish‐labeled Streptomycin working solution. Subsequently, the sections were stained with DAB chromogenic solution for 3–5 min, followed by a 30‐s hematoxylin stain. Finally, the samples were rendered transparent, air‐dried, sealed with neutral resin, and examined under an optical microscope.

### 2.6 Multiple fluorescence immunohistochemistry

Per Absin reagent instructions, clinical sample tissue sections were incubated at 60°C for 1 h, dewaxed with xylene and ethanol, washed with TBST, subjected to antigen retrieval in a microwave, rinsed with TBST, and had catalase added to block endogenous peroxidase. After incubating TBST for 10 min at room temperature, it was rinsed. Normal sheep serum working solution was then added and incubated for 30 min at 37°C for blocking. The primary antibody was added and incubated overnight at 4°C. The next day, the second antibody working solution was added and incubated for 10 min, then rinsed with TBST. The sample was then incubated with fluorescent staining amplification signal at a dilution of 1:100. Staining was confirmed using fluorescence microscopy, and additional staining could be added if needed. The slides underwent a thermal repair process to remove the tissue‐bound primary and secondary antibody complexes prior to additional labeling. This procedure was repeated iteratively until the clinical tissue slides were diluted to a ratio of 1:100 with all markers and spectral DAPI (Absin Inc.). Subsequently, an anti‐fluorescence quenching reagent was applied, and the slides were sealed with a cover glass. Images of each case were then captured using the Olympus panorama scanner.

### 2.7 Enzyme‐linked immunosorbent assay

Following the ELISA kit instructions, colon tissues were rinsed, weighed, and sliced. The tissues were then homogenized and centrifuged to collect the supernatant. The standard product and samples were added to the plate, with blank holes set and HRP labeled detection antibody added to both blank and sample holes. Sealing holes with plate membranes, incubating at 37°C for 60 min, adding substrates A and B, incubating at 37°C for 15 min without light, adding stop solution, detecting OD values at 450 nm wavelength, and calculating contents of INF‐γ, TNF‐α, IL‐4, and IL‐10 in tissue supernatant.

### 2.8 Real‐time fluorescence quantitative PCR

Total RNA was extracted from colon tissues or cells using the Trizol method, and its concentration was measured with an ultramicro nucleic acid protein detector. Reverse transcription and RT‐qPCR were conducted following kit instructions, and data analysis was done using the 2^−ΔΔCt^ method or the relative quantitative ratio method with a double standard curve (Table S1 for primer sequences).

### 2.9 Western‐Blot

Following dissociation of the tissue samples, the supernatant was collected through centrifugation at 12,000 rpm at 4°C for 15 min. Protein concentration was assessed using the BCA method, and the volume of each protein sample (40 mg for cell systems and 100 mg for colon tissue sample systems) was calculated. Subsequently, sample loading, electrophoresis, and membrane transfer procedures were conducted. The PVDF membrane was blocked using a 5% skim milk solution and subsequently incubated at 4°C overnight following TBST washing. After washing, the secondary antibody was incubated on a room temperature shaker for 1 h. The PVDF membrane was then developed using an ECL luminescent solution in a ChemiDoc TMXRS+ imager. The Image J software measured the gray value of each strip to determine the relative expression level of the target protein compared to the reference protein (Table S2 for antibody names and concentrations).

### 2.10 Immunofluorescence

Place the sliver in a 24‐well plate, add organoid suspension, and incubate at 37°C for 15 min. Once the matrix glue solidifies, add the culture solution. After 96 h, remove the culture medium, wash with PBS, and fix with 4% paraformaldehyde at 4°C overnight. Remove the fixative and add pre‐cooled IF buffer (0.2% Tritonx‐100 + PBS + 0.1% TWEEN‐20) three times for 5 min each. Permeate with 0.5% Tritonx‐100 for 20 min, then rinse with IF buffer three times for 5 min each. Blocked with 1% BAS for 30 min, then added primary antibody and incubated at 4°C overnight. Washed with pre‐cooled IF buffer three times for 5 min each. Added fluorescent secondary antibody and incubated for 1 h at room temperature. Stained nucleus with DAPI dye for 3 min, then rinsed with IF buffer three times for 5 min each. Apply anti‐fluorescence quench agent, remove excess, and seal slide with nail oil. Dry for 1 h in darkness, then photograph under fluorescence microscope.

### 2.11 Statistical analysis

Statistical software SPSS 26.0 was used for data analysis. Data conforming to normal distribution were represented by $\bar{x}$ ± s. Comparison between two groups was analyzed by *t* test or *t*′ test, and comparison between multiple groups was analyzed by one‐way variance (ANOVA). The statistical data disobeys normal distribution were represented by median (*M*) and interquartile distance (IQR). Wilcoxon rank sum test or Mann–Whitney *U* test was used for comparison between two groups, and Kruskal–Wallis *H* test was used for comparison between multiple groups. *p* < 0.05 was statistically significant.

## 3 RESULTS

### 3.1 Clinical research

**FIGURE 1:**
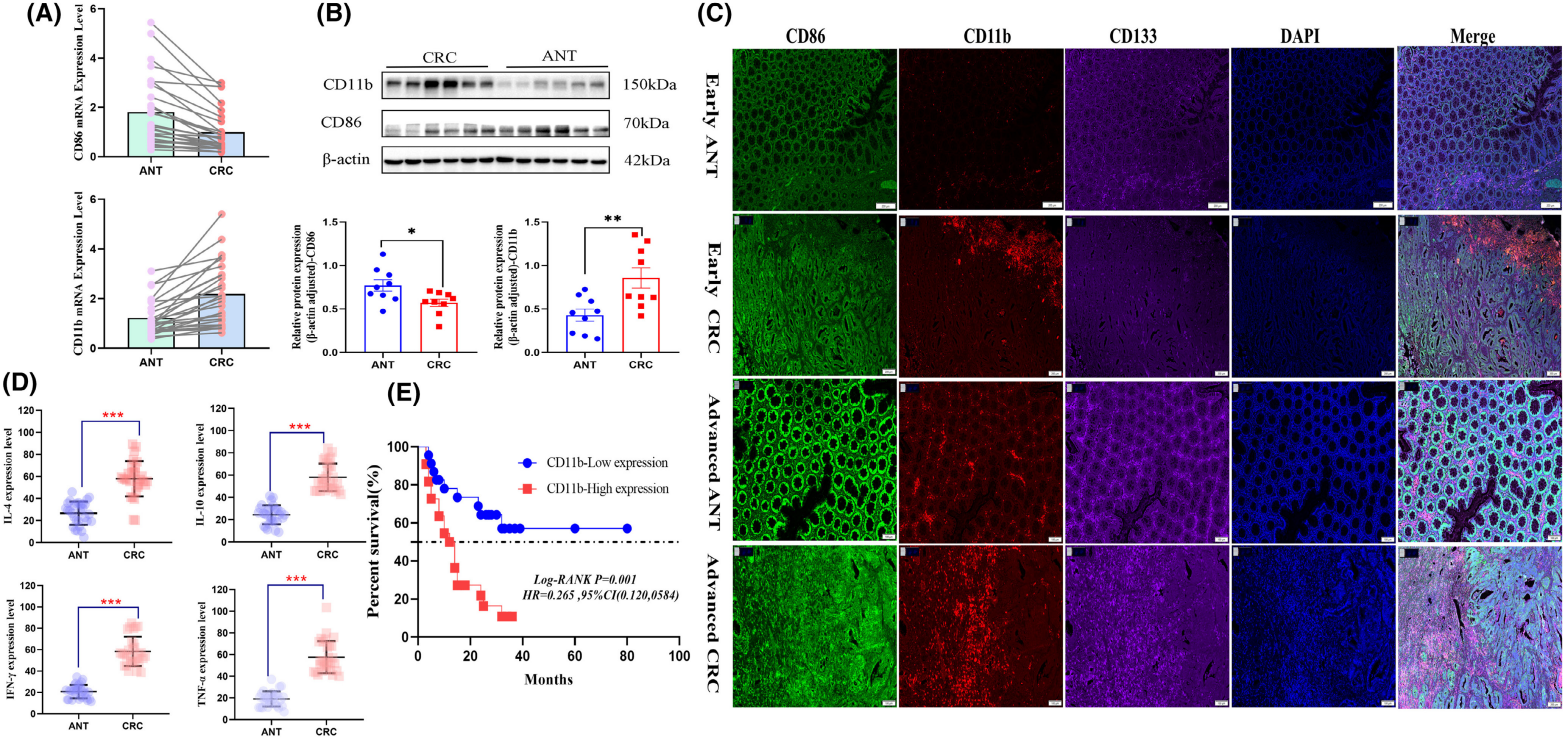
Expression changes of CD86/CD11b in CRC tissues/adjacent normal tissues and their relationship with prognosis. (A) mRNA levels of CD86/CD11b in CRC tissues and adjacent normal tissues were detected using qRT‐PCR (*n* = 27); (B) Protein levels of CD86/CD11b in CRC tissues and adjacent normal tissues were detected by Western blotting (*n* = 9); (C) Protein levels of CD86, CD11b, and CD133 were detected by multiple fluorescence immunohistochemistry in adjacent normal tissues (ANT) and CRC tissues. CD86 was labeled with green fluorescence, CD11b with red fluorescence, CD133 with purple fluorescence, and DAPI with blue fluorescence (magnification ×200); (D) Changes in the levels of IL‐4, IL‐10, IFN‐γ, and TNF‐α in different intestinal tissues of CRC patients were measured using ELISA (*n* = 30); (E) Kaplan–Meier survival analysis was performed to evaluate the prognostic value of CD11b (high expression defined as > median (AOD value) of CD11b protein expression, low expression defined as < median) in CRC patients (*n* = 65). *p < 0.05,**p < 0.01, ***p < 0.001.

In this research, surgical samples from CRC patients were initially analyzed, including both CRC tissues and adjacent normal tissues. Table 1 displays the baseline clinical and pathological characteristics of the patient cohort. The results demonstrated CD11b expression was significantly higher in CRC tissues than adjacent normal tissues, while CD86 expression was decreased. Additionally, elevated levels of IL‐4, IL‐10, IFN‐γ and TNF‐α were observed in CRC tissues (Figure 1A,B,D). Multiple staining analysis revealed a significantly higher expression of CD11b in the early and advanced CRC tissues compared with the adjacent normal tissues (Figure 1C). This elevated expression was predominantly observed in the mesenchymal cell region. In contrast, the expression of CD86 was predominantly localized in the adenoepithelial region, with no significant changes in the early CRC tissues and decreased in the advanced CRC. The expression of CD11b in the advanced CRC was mainly overlapped with CD133‐positive areas. These findings suggested that CD11b might serve as a potentially more sensitive marker in CRC microenvironment.

**TABLE 1:** Baseline clinicopathological characteristics of the patients.

Patient characteristics	Number of patients (*N* = 65)	Percentage(%)
*Gender*		
Male	26	40.00
Female	39	60.00
*Age* (*year*)		
<60	38	58.46
≥60	27	41.54
*Histological type*		
Adenocarcinoma	53	81.54
Non‐adenocarcinoma	12	18.46
*Differentiation*		
High differentiation	30	46.15
Moderately differentiated	22	33.85
Poorly differentiated	13	20.00
*TNM staging*		
I	8	12.30
II	39	60.00
III	9	13.85
IV	9	13.85
*Depth of intestinal wall infiltration*		
Mucous membrane layer	9	13.85
Muscle layer	9	13.85
Serous layer	44	67.69
Outer layer	3	4.61
*Tumor size*		
<5 cm	32	49.23
≥5 cm	33	50.77
*Lymph node metastasis*		
Without	40	61.54
With	25	38.46
*CEA*		
<5	35	53.85
≥5	30	46.15
*CA199*		
<37	46	70.77
≥37	19	29.23
*Survival state*		
Death	32	49.23
Survival	33	50.77
*CD11 expression*		
Low expression	33	50.77
High expression	32	49.23

Immunohistochemistry was performed on paraffin sections from CRC patients to evaluate CD11b expression. The results indicated that CD11b expression was correlation with the tumor size (*p* = 0.018, Table 2). Cox regression analysis (Table 3) suggested that CD11b could serve as a predictor of survival prognosis. CRC patients with high levels of CD11b infiltration showed a higher risk of recurrence compared to those with low levels of infiltration (*p* < 0.05). This implies that patients with low levels of CD11b expression have a more favorable survival prognosis (Figure 1E). However, due to the small sample size, further follow‐up studies are warranted to confirm these conclusions.

**TABLE 2:** Correlation of CD11b expression with clinicopathological information characteristics.

Clinicopathological parameters	*n*	CD11b expression		*p*
		Low expression	High expression	
*Gender*				
Male	26	10	16	0.105
Female	39	23	16	
*Age* (*year*)				
<60	27	16	11	0.248
≥60	38	17	21	
*Histological type*				
Adenocarcinoma	53	24	29	0.063
Others	12	9	3	
*Degree of differentiation*				
High differentiation	30	17	13	0.241
Moderately differentiation	22	8	14	
Poorly differentiation	13	8	5	
*Depth of tumor invasion*				
T1	9	3	6	0.148
T2	9	6	3	
T3	44	24	20	
T4	3	0	3	
*Tumor size*				
<5 cm	32	21	11	0.018*
≥ 5 cm	33	12	21	
*TNM staging*				
I	8	5	3	0.213
II	39	18	21	
III	9	7	2	
IV	9	3	6	
*Lymph node metastasis*				
Without	40	20	20	0.875
With	25	13	12	
*CEA*				
<5 ng/mL	21	19	16	0.540
≥5 ng/mL	24	14	16	
*CA199*				
<37 U/mL	46	24	22	0.724
≥37 U/mL	19	9	10	

* *p* < 0.05.

**TABLE 3:** Multivariate analysis of predictive survival factors.

Clinicopathological paramete	Hazard ratio	95.0% CI for Exp(B)		*p*
		Lower	Upper	
*Gender*				
Male	1.00	0.301	1.712	0.456
Female	0.719			
*Age* (*year*)				
<60	1.00	0.237	1.363	0.206
≥60	0.569			
*Histological type*				
Adenocarcinoma	1.00	1.473	0.526	0.461
Others	0.461			
*Degree of differentiation*				
High differentiation	1.00	0.843	2.520	0.178
Moderately differentiation Poorly differentiation	1.457			
*Depth of tumor invasion*				
T1 and T2	1.00	0.586	1.887	0.867
T3 and T4	1.051			
*Tumor size*				
<5 cm	1	0.294	1.952	0.565
≥5 cm	0.758			
*TNM staging*				
I and II	1.00	1.033	2.941	0.037^a^
III and IV	1.743			
*Lymph node metastasis*				
Without	1.00	0.156	0.950	0.038^a^
With	0.385			
*CEA*				
<5 ng/mL	1.00	0.422	2.809	0.860
≥5 ng/mL	1.085			
*CA199*				
<37 U/mL	1.00	0.762	4.496	0.174
≥37 U/mL	1.851			
*CD11b*				
Low expression	1.00	1.132	7.181	0.026^a^
High expression	2.851			

Abbreviations: CD11b, cluster of differentiation 11b; CI, confidence interval.

^a^ *p* <0.05.

### 3.2 Animal experiments

**FIGURE 2:**
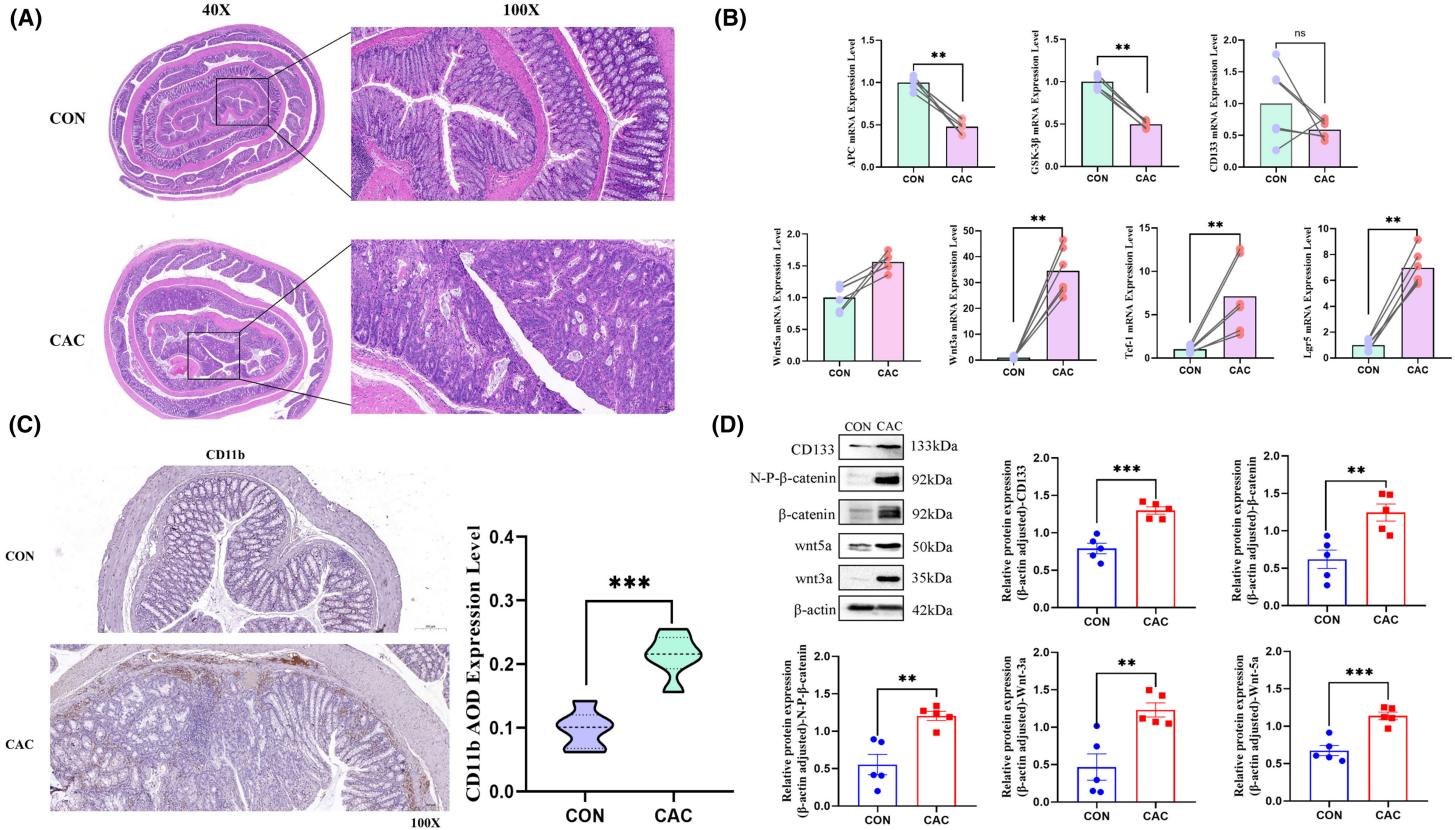
Changes in CD11b expression and of Wnt signaling pathway‐related proteins in the colon of CAC model mice. (A) The colon tissues of CAC model mice and control mice (magnification HE X50); (B) Relative mRNA expression levels of APC, GSK‐3β, CD133, Lgr5, Wnt3a, Wnt5a, and Tcf‐1 in the colon of each group of mice; (C) Immunohistochemistry showed the protein expression of CD11b in the colon; (D) Changes in protein expression of CD133, β‐Catenin, N‐P‐β‐Catenin, Wnt3a, and Wnt5a in the colon of each group (n = 6). ns: Not significant *p* > 0.05, **p < 0.01, ***p < 0.001.

Colitis‐associated cancer mice exhibited the colonic adenocarcinoma with gland structural disarray and size heterogeneity, disruption of epithelial cell nuclear orientation, and deep stain. Some glands also displayed adenomatous alterations. Moreover, the mRNA levels of Lgr5, Wnt3a, and Tcf‐1 were significantly elevated in the CAC group compared with the control group (*p* < 0.01), while the level of APC and GSK‐3βmRNA decreased (*p* < 0.01; Figure 2B). The study found significant molecular changes in adenocarcinoma tissues from a CAC mouse model, including increased expression of Wnt3a and Wnt5a proteins, total β‐catenin protein, and activated β‐catenin. The tumor stem cell markers CD133 and Lgr5 were also significantly elevated (Figure 2D, *p* < 0.01). Due to the lack of Paneth cells secreting Wnt3a in the colon mucosa, the source of Wnts mainly depends on the stromal cells around the colonic crypt stem cells. The results suggested that the abnormal activation of Wnt signaling pathway from other cell sources in the CAC tumor microenvironment might provide support for the occurrence of epithelial adenocarcinoma; meanwhile, the stromal cell CD11b expression in the CAC mouse adenocarcinoma region was significantly increased (Figure 2C), suggesting that it may be involved in the above pathological mechanism.

### 3.3 Cell experiments

**FIGURE 3:**
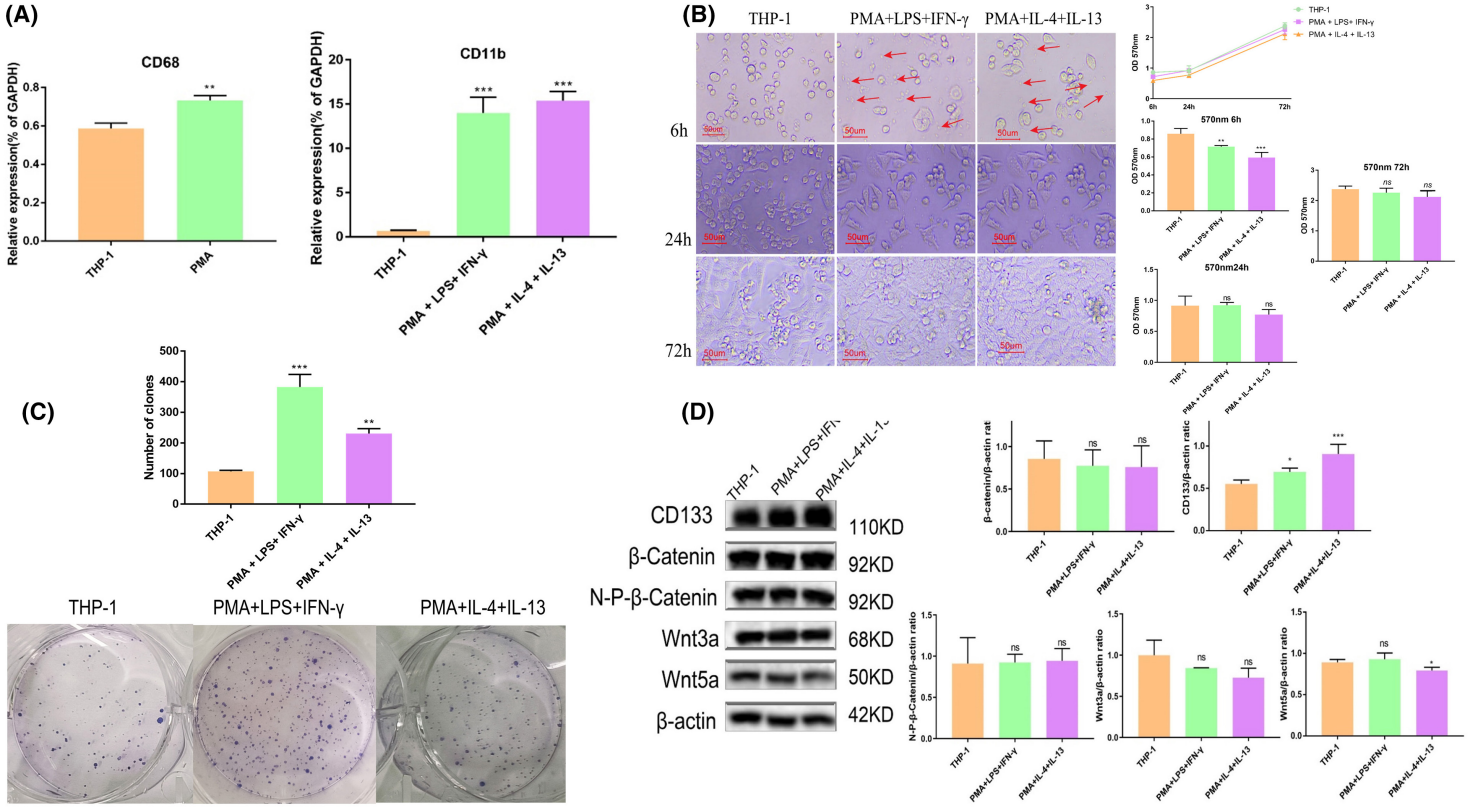
Induction of THP‐1 cell differentiation and the effect of induced conditioned medium on HCT116 cells. (A) PMA stimulation induces differentiation of THP‐1 cells into macrophages, further promoting increased expression of CD11b. (B) The impact of conditioned medium from induced macrophages on the morphology and growth of HCT116 cells. (C) The influence of conditioned medium on colony formation of HCT116 cells. (D) Changes in the expression of Wnt3a, Wnt5a, CD133, and β‐catenin/N‐P‐β‐catenin proteins in HCT116 cells after co‐culture. Error bars represent mean ± standard deviation (*n* = 3). ns: Not significant (*p* > 0.05), *p < 0.05, **p < 0.01, ***p < 0.001.

In vitro, both PAM+LPS + IFN‐γand PAM+IL4 + IL13 stimulation could induce CD11b mRNA level markedly increased in THP‐1 cells (Figure 3A, *p* < 0.001). Then the two groups of conditional culture medium of induced THP‐1 cells were co‐cultured with HCT116 cells respectively. After 6 h of co‐culture, partial death of HCT116 cell was observed under the microscope. After 24 h, the remaining HCT116 cell formed pseudopodia and aggregated into cell clusters, and then reached 90–100% fusion after 72 h, which was no different from the control group cells. The MTT analysis was consistent with the above microscope observation. The growth rate of HCT116 cell was significantly lower than that of the control group cell at 6 h after co‐culture with the above conditional medium, but there was no significant difference at 24 and 72 h (Figure 3B). Further, HCT116 colony formation was significantly increased by co‐culturing for 72 h (Figure 3C). The western blot results showed that only CD133 protein expression was significantly higher than that of the control group at 72 h, while Wnt3a, β‐catenin, and N‐P‐β‐catenin did not change obviously (Figure 3D). These results suggested that macrophages with high expression of CD11b could promote the proliferation of tumor stem cells. Among them, PMA + IL‐4 + IL‐13 induced medium caused Wnt5a protein expression decreased in HCT116 cells, which may be related to the differentiation‐related functions.

### 3.4 Organoid experiments

**FIGURE 4:**
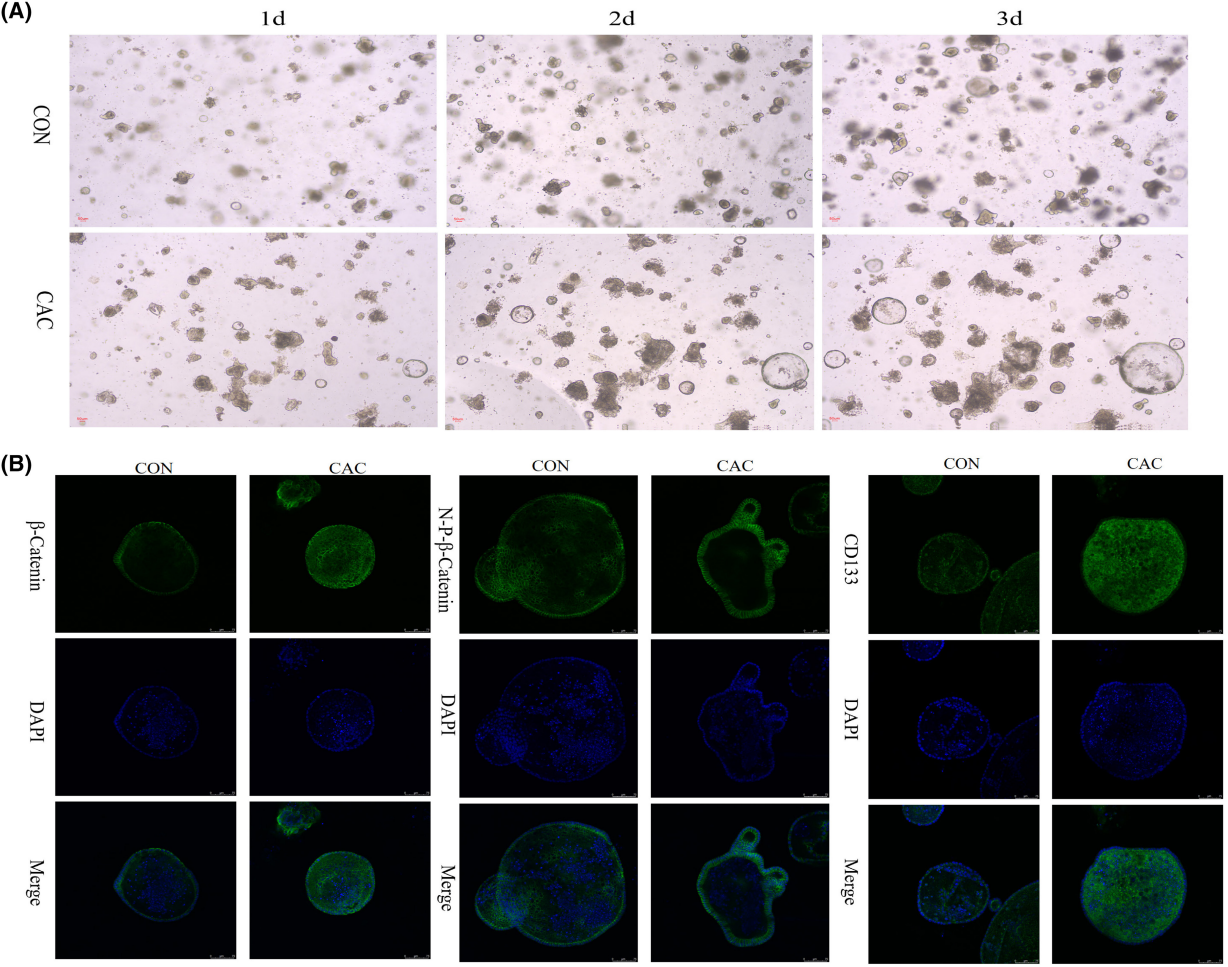
Colonic organoids culture from CAC mice and the expression changes of β‐Catenin, N‐P‐β‐Catenin, and CD133 proteins. (A) Morphological changes in the growth of colon organ culture from control and CAC mice (1–3 days) (magnification X40); (B) Immunofluorescence detection of the expression changes of β‐Catenin, N‐P‐β‐Catenin, and CD133 in colon organ culture (magnification ×200). *n* = 3.

Finally, colon crypts were isolated for 3D culture to form colonic organoids. The morphology of the organoids derived from the CAC mice presented more cystic changes of different sizes, mixed with some adherent cells (possibly the tumor associated stromal cells) in the matrigel (Figure 4A). Immunofluorescence staining showed that CD133 and β‐catenin/N‐P‐β‐catenin fluorescence intensity were significantly increased (Figure 4B). These data suggested that crypt stem cells may be an important source of tumor stem cells, and the Wnt signal activation produced by stem cells was related to the increased CD133 expression. Nevertheless, these results are preliminary and additional comprehensive investigations are necessary to understand the mechanisms.

## 4 DISCUSSION

The etiology of CRC includes genetic predisposition, dietary patterns, lifestyle factors, exposure to carcinogens, gastrointestinal disorders, and other variables.^14^, ^15^, ^16^ The transition from low‐grade dysplasia to high‐grade dysplasia and eventually to malignant neoplasms generally spans a period of 5–10 years.^17^, ^18^Scientific evidence suggests a correlation between an upsurge in M2 macrophages within CRC and unfavorable patient prognosis.^19^ M2 macrophages are known to secrete a variety of growth factors and regulatory molecules, thereby facilitating angiogenesis, extracellular matrix remodeling, immune evasion, and tumor expansion.^20^, ^21^, ^22^, ^23^, ^24^ The balance between M1 and M2 phenotypes in CRC is modulated by elements within the tumor microenvironment.^25^ Comprehensive research underscores that CD11b expression is amplified in immune cells infiltrating a variety of solid tumors. Crucially, the infiltration of CD11b‐positive cells into tumors may reshape the tumor microenvironment, thereby influencing tumor growth and dimensions.^26^, ^27^ Clinical data in our studies demonstrate a correlation between the extent of CD11b infiltration in CRC and tumor size. Moreover, patients exhibiting high levels of CD11b infiltration are at an elevated risk of recurrence compared to those with lower CD11b infiltration. Furthermore, increased levels of IL‐4, IL‐10, IFN‐γ, and TNF‐α indicated a potential dysfunction of macrophages. The self‐renewal capability of CSCs in the context of the tumor stem cell theory has been implicated in the progression, recurrence, metastasis, and proliferation of tumors.^28^ Additionally, a body of research has demonstrated that CD133 serves as a valuable independent prognostic indicator in individuals with colorectal cancer, with higher levels of CD133 expression being associated with advanced tumor stages and liver metastasis.^29^, ^30^ Our findings indicated that CD133 expression was obviously elevated in the advanced CRC tissues and the location mainly coincide with CD11b positive area. It is hypothesized that CD11b may serve as a more sensitive indicator within the tumor microenvironment of colorectal cancer patients, with its elevated expression showing a positive correlation with the tumor stem cell marker CD133.

The sustained activation of the Wnt signaling pathway is postulated to be linked with tumorigenesis and is perceived as the precipitating event of colorectal cancer.^31^, ^32^ In the CAC model mice, we observed Wnt3a, Wnt5a, β‐catenin, and non‐phosphorylated (activated) β‐catenin were markedly upregulated in early adenocarcinoma tissues. The expression of CD133 and CD11b also increased with Wnt signal activation. Given that the colonic mucosa is devoid of Paneth cells that secrete Wnt3a, the source of Wnts in the colon primarily relies on the stromal cells surrounding the intestinal crypt stem cells. These results suggested that the aberrant activation of the Wnt signaling pathway from the CAC tumor microenvironment lends support for the development of epithelial adenocarcinoma. The expression of CD11b in stromal cells in the adenocarcinoma region might assume a crucial role in the pathological mechanism.

Although it is well‐established that TAMs are abundant within the tumor microenvironment, the complex mechanisms that regulate the tumor‐promoting functions of TAMs remain unclear. Some studies found that CD11b/CD18 was not required for adhesion to endothelium or trafficking into tumors.^33^ In contrast, CD11b/CD18 showed to mediate macrophage adhesion, migration, and accumulation during inflammation, CD11b signaling modulated neovascularization and promoted anti‐tumor immune responses.^34^ We reported here that HCT116 cells exposed to the high CD11b expression of induced THP‐1 cells culture medium exhibited significant augment of colony number at 72 h. On this basis, CD133 protein expression was also increased in HCT116 cells. These findings suggest that macrophages expressing high levels of CD11b may facilitate the growth of cancer stem cells. Furthermore, treating THP‐1 cells with PMA + IL‐4 + IL‐13 for 72 h resulted in a decrease in Wnt5a protein expression in HCT116 cells, which may be associated with its involvement in tumor cell differentiation‐related functions.

We isolated colonic crypt stem cells and cultured the mini gut to observe the potential activation role of the Wnt signaling pathway in promoting tumor stem cell proliferation. Previous research has found that intestinal organoid in vitro has characteristics similar to intestine in vivo. It is considered the best model for studying stem cell proliferation and differentiation as well as the occurrence of intestinal tumors. Cell proliferation and crypt formation play a crucial role in the development of colorectal adenomas and proliferative polyps.^35^, ^36^ We found that the colonic organoids derived from the CAC mice showed tumor‐like characteristics, presenting a multicystic spherical shape. Immunofluorescence results showed CD133 and β‐catenin/N‐P‐β‐catenin intensity markedly increased. We speculate that crypt stem cells may be an important source of tumor stem cells, and the Wnt signal activation mainly produced by stem cells is related to the increased expression of CD133. These are only preliminary results, and further in‐depth research is needed.

## AUTHOR CONTRIBUTIONS

**Junyu Ke:** Data curation (equal); writing – original draft (equal). **Guirong Chen:** Writing – original draft (equal). **Yihui You:** Formal analysis (lead); validation (supporting). **Qinghua Xie:** Formal analysis (equal); validation (equal). **Zheng‐lin Liu:** Data curation (equal); formal analysis (equal); investigation (equal); validation (equal). **Chunhui Song:** Formal analysis (equal); validation (equal). **Yanqiu Zheng:** Formal analysis (equal); validation (equal). **Zejun Shan:** Writing – review and editing (equal). **Jinbin Song:** Formal analysis (equal); validation (equal). **Zhangyu Jiang:** Validation (equal). **Haiyan Wang:** Validation (equal). **Qun Du:** Methodology (equal); resources (equal). **Yongqiang Wu:** Project administration (equal); resources (equal). **Xin‐lin Chen:** Data curation (equal); resources (equal); writing – review and editing (equal). **Yanwu Li:** Funding acquisition (equal); resources (equal); writing – review and editing (equal).

## FUNDING INFORMATION

This work was supported by the National Natural Science Foundation of China (Grant Nos. 82274600, and 81774451); Guangdong Provincial Bureau of Traditional Chinese Medicine research project (Nos. 20231396 and 20241382); Science and technology innovation special topic of Maoming City (Nos. 2023SZX027, 2023SZY011 and 2023SZX017).

## CONFLICT OF INTEREST STATEMENT

The authors declare that this study was conducted in the absence of any commercial or financial relationships that could be construed as potential conflicts of interest.

## ETHICS STATEMENT

This study was conducted in accordance with the Declaration of Helsinki and were approved by the Ethics Committee of Gaozhou Hospital of Traditional Chinese Medicine Affiliated to Guangzhou University of Chinese Medicine (reference number: yxllyjky20200518). Meanwhile, The animal experiment was approved by the Animal Ethics Committee of Guangzhou University of Traditional Chinese Medicine (No. 20210329005). Animal quality certificate: No. 44007200090003.

## Supporting information


Table S1:

Table S2:

## Data Availability

The datasets generated for this study are available upon request to the corresponding author.
