# Supplementary material for: CD11b/CD86 involved in the microenvironment of colorectal cancer by promoting Wnt signaling activation
Source: Cancer Med. 2024 Sep 20;13(18):e70245. doi: 10.1002/cam4.70245 (PMC11413919; doi:10.1002/cam4.70245)
Supplement: Supplementary file 1 — Table S1: [file CAM4-13-e70245-s002.docx]

| Gene name | Forward (5, 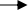 3,) | Reverse (5,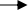3,) |
| --- | --- | --- |
| CD80(human) | AAACTCGCATCTACTGGCAAA | GGTTCTTGTACTCGGGCCATA |
| CD86(human) | CTGCTCATCTATACACGGTTACC | GGAAACGTCGTACAGTTCTGTG |
| CD11b(human) | GCCTTGACCTTATGTCATGGG | CCTGTGCTGTAGTCGCACT |
| CD163(human) | TTTGTCAACTTGAGTCCCTTCAC | TCCCGCTACACTTGTTTTCAC |
| CD14(human) | ACGCCAGAACCTTGTGAGC | GCATGGATCTCCACCTCTACTG |
| CD68(human) | CTTCTCTCATTCCCCTATGGACA | GAAGGACACATTGTACTCCACC |
| Lgr5(human) | GAGTTACGTCTTGCGGGAAAC | TGGGTACGTGTCTTAGCTGATTA |
| Wnt3a(human) | AGCTACCCGATCTGGTGGTC | CAAACTCGATGTCCTCGCTAC |
| CD133(human) | AGTCGGAAACTGGCAGATAGC | GGTAGTGTTGTACTGGGCCAAT |
| Wnt5a(human) | GCCAGTATCAATTCCGACATCG | TCACCGCGTATGTGAAGGC |
| APC(human) | AAGCATGAAACCGGCTCACAT | CATTCGTGTAGTTGAACCCTGA |
| Tcf-1(human) | TTGATGCTAGGTTCTGGTGTACC | CCTTGGACTCTGCTTGTGTC |
| GSK-3β(human) | GGCAGCATGAAAGTTAGCAGA | GGCGACCAGTTCTCCTGAATC |
| GAPDH(human) | ACAACTTTGGTATCGTGGAAGG | GCCATCACGCCACAGTTTC |
| APC(mouse) | GAGGAGGAAACTGGAGGAA | GGCTTAAAACTGGGGTCTG |
| GSK-3β(mouse) | CCCACCATCACCATTAAGA | AATCCACCTTGCTTTCCA |
| CD133(mouse) | ACTGGGGCTGTGTGGAAAG | GCATTGAAGGTATCTTGGGTCTC |
| Lgr5(mouse) | ACATTCCCAAGGGAGCGTTC | ATGTGGTTGGCATCTAGGCG |
| Wnt3a(mouse) | GCCCTTCTTCCCACTTGT | AAACCCAGCCAGTACCATC |
| Wnt5a(mouse) | GGCAGGACTTTCTCAAGGA | CGGAACTGGTACTGGCAT |
| Tcf-1(mouse) | CCACTCTACGAACATTTCAGCA | ACTGGGCCAGCTCACAGTA |
| GAPDH(mouse) | CAAGGCTGTGGGCAAGGTCATCC | TTTCTCCAGGCGGCAGGTCAGAT |

**Table 1. Primer sequence of targeted genes**
