# Supplementary material for: CD11b/CD86 involved in the microenvironment of colorectal cancer by promoting Wnt signaling activation
Source: Cancer Med. 2024 Sep 20;13(18):e70245. doi: 10.1002/cam4.70245 (PMC11413919; doi:10.1002/cam4.70245)
Supplement: Supplementary file 2 — Table S2: [file CAM4-13-e70245-s001.docx]

**Table 2. Antibody name and concentration**

| Antibodies | Dilutions | Sources | Cat.NO. |
| --- | --- | --- | --- |
| CD11b | 1:200(WB) | Abcam，USA | Ab6586 |
| β-Catenin | 1:1000(WB) | CST，USA | 8840S |
| N-P-β-Catenin | 1:1000(WB) | CST，USA | 8814S |
| Wnt5a | 1:1000(WB) | Proteintech | 55184-1-AP |
| Wnt3a | 1:500(WB) | Proteintech | 26744-1-AP |
| CD133 | 1:1000(WB) | Abcam，USA | Ab19898 |
| CD86 | 1:500(IHC);1:1000(WB) | Abcam，USA | Ab243887 |
| CD11b | 1:1000(IHC/WB); | Abcam，USA | Ab52478 |
| β-actin | 1:5000(WB) | Abcam，USA | Ab179467 |
